# Effects of Different Forms of Milk Thistle Supplementation in Rabbit Diets on Stress-Induced Physiological Responses

**DOI:** 10.3390/ani15243582

**Published:** 2025-12-12

**Authors:** Hana Dočkalová, Daria Baholet Bátik, Pavel Horký, Marie Balabánová, Andrej Bátik, Jaroslav Ondráček, Adéla Dokoupilová

**Affiliations:** 1 Department of Animal Nutrition and Forage Production, Faculty of AgriSciences, Mendel University in Brno, 61300 Brno, Czech Republic; hana.dockalova@mendelu.cz (H.D.); daria.baholet@mendelu.cz (D.B.B.); pavel.horky@mendelu.cz (P.H.); marie.balabanova@mendelu.cz (M.B.); 2 Department of Morphology, Physiology and Animal Genetics, Faculty of AgriSciences, Mendel University in Brno, 61300 Brno, Czech Republic; 3 BIOKRON s.r.o., 66456 Blučina, Czech Republic; biokron@biokron.cz; 4 Department of Ethology and Hobby Breeding, Czech University of Life Sciences Prague, 16521 Prague, Czech Republic; dokoupilova@af.czu.cz

**Keywords:** rabbit welfare, thermal stress, noise stress, milk thistle supplementation, natural feed additives, growth performance

## Abstract

This study evaluated the effects of thermal and noise stress on rabbits (90 HYLA hybrids), focusing on physiological responses, production performance, and welfare. Three dietary treatments were compared: a control diet (C), a diet supplemented with milk thistle seed cake (SMT), and a diet containing fermented milk thistle seed cake (FMT). The aim was to assess whether these feed additives could mitigate the adverse effects of environmental stress. Health status was monitored through clinical observation and analysis of biochemical indicators, including enzymes, lipid and protein metabolism markers, and total antioxidant status. Thermal stress at the onset of fattening influenced average daily gain, feed intake, and carcass yield, while dietary intervention modulated these effects. The FMT group demonstrated the lowest feed intake and most efficient feed conversion during later growth stages, but had lower slaughter weight compared with the control. In contrast, rabbits fed the SMT diet achieved the highest slaughter weight and superior growth efficiency. These findings suggest that milk thistle, particularly in combination with probiotic fermentation, can enhance feed utilization, growth performance, and stress resilience in rabbits, contributing to more sustainable and welfare-oriented production systems.

## Simple Summary

Rabbits raised for meat production are frequently exposed to environmental stressors such as heat and noise, which can negatively impact their health, growth, and overall production efficiency. This study evaluated whether dietary supplementation with milk thistle seed cake or its fermented form could alleviate these stress effects. Ninety rabbits were assigned to one of three diets: a standard control diet, a diet supplemented with 2% milk thistle, or a diet containing a fermented substrate with 1% milk thistle. Growth performance, feed intake, and biochemical blood parameters were closely monitored. The results demonstrated that milk thistle improved growth and feed efficiency, particularly during the early stages of fattening. Rabbits fed fermented milk thistle consumed less feed and exhibited better feed conversion, but gained slightly less weight overall. The group receiving 2% milk thistle achieved the highest body weight and the best growth performance. No health issues or mortality were observed during the study. These findings suggest that milk thistle, especially in its natural form, can support rabbit health and productivity under stress, offering a natural approach to enhance welfare and sustainability in rabbit production.

## 1. Introduction

Rabbit meat is valued for its nutritional properties and is increasingly popular in human diets, driving the expansion of rabbit production worldwide [1,2,3]. Rabbits have a short life cycle, are highly prolific, possess a short gestation period, and exhibit high feed conversion efficiency [4]. Rabbit meat is considered a healthy food, being rich in protein while low in fat, cholesterol, and sodium [5], making it particularly suitable for children and the elderly [6]. Unlike other livestock, rabbits lack well-developed sweat glands except on the lips and inguinal region, which limits their ability to regulate body temperature [7].

Heat stress in rabbits is influenced by multiple factors, including high ambient temperature, humidity, radiant heat, and air velocity, with ambient temperature being the most critical factor affecting welfare and performance [8]. The physiological body temperature of healthy rabbits is 39 ± 0.5 °C, the thermoneutral zone is 20 ± 5 °C, and the optimal environmental humidity is 60 ± 5% [9,10,11]. For 6- to 12-week-old rabbits, the most favorable temperature range has been reported as 15–18 °C [12].

Rising ambient temperatures have pronounced physiological and productive effects. An increase from 20 to 30 °C accelerates respiration (from ~40 to 200 breaths per minute) and elevates body temperature up to 43 °C. Temperatures above 30 °C reduce reproductive performance (e.g., litter size) and lactation, while temperatures exceeding 35 °C significantly increase mortality [7]. Heat stress can also reduce daily weight gain by 20–25%, impair feed conversion by 8–15%, increase mortality by 9–12%, decrease reproductive performance by 6–10%, and negatively affect meat quality and carcass traits [2]. According to the authors of [13], heat stress above 15 °C decreases milk production and reduces feed and water intake in does, whereas offspring appear more resilient to elevated temperatures than their mothers.

Nutritional management provides a practical strategy to mitigate the effects of heat stress. Adjustments in diet composition, as well as supplementation with vitamins, minerals, electrolytes, or functional bioactive compounds, can alleviate the detrimental impacts of heat stress in rabbits [2]. Noise is another important stressor in rabbit husbandry, capable of triggering startle responses and abnormal behaviors that may result in traumatic limb injuries, increased aggression, impaired reproductive and maternal performance, and reduced meat production [10,14].

Laboratory monitoring of noise frequency has shown that rabbits are exposed to frequencies between 10 and 40/60 kHz in their housing environment due to cage interactions, such as trampling on different materials [15]. Rabbits can hear sounds up to 42 kHz, and noise in rabbit housing should remain below 60 dB during quiet periods and below 70 dB during work activities, with peaks not exceeding 85 dB [16]. High sound levels from reproductive equipment may have adverse rather than preventive effects. Optimal biological functioning occurs when animals are maintained in appropriate environments, ensuring maximal performance, welfare, and quality of life [17]. To prevent stress-related health disorders, dietary supplementation with *Silybum marianum* has shown potential, as confirmed in previous studies on horses [18].

Milk thistle (*Silybum marianum* L.), a member of the Asteraceae family native to arid and semi-arid Mediterranean regions, is an annual plant rich in silymarin flavonolignans with hepatoprotective and chemoprotective properties; it is present throughout the plant but occurs at the highest concentrations in seeds. Milk thistle can be administered as whole or ground seeds, seed oil, or seed cakes (residues after oil extraction) to improve animal nutrition [19,20].

The main bioactive component derived from milk thistle seed cakes is silymarin, a complex of antioxidant flavonolignans (65–85%) [21]. This complex contains silibinin (silybins A and B; ~50%), isosilybin (isosilybins A and B; ~5%), silychristin (~20%), and silydianin (~10%) [22]. Silibinin has been reported to exhibit anti-inflammatory and hepatoprotective effects in experimental rats with induced preeclampsia [23]. Among other flavonoids, taxifolin contributes significantly to overall antioxidant capacity [24]. This flavonolignan complex protects liver cells by stabilizing membrane permeability, inhibiting lipid peroxidation, and preventing hepatic glutathione depletion [25]. Additionally, silymarin inhibits enzymes responsible for reactive oxygen species (ROS) production, reducing free radical formation and enhancing mitochondrial integrity under stress conditions [21].

The biological activity of *Silybum marianum* flavonolignans is closely related to the organism’s antioxidant capacity, enabling it to cope with environmental stress. Heat stress is a major challenge in rabbit production, negatively affecting feed intake, physiological homeostasis, reproductive performance, and immune function [2]. For example, supplementation with milk thistle extract at dietary levels of 10 or 15 g/kg has been shown to enhance growth performance, feed utilization, dressing percentage, hemato-biochemical parameters, immunity, and redox balance in heat-stressed growing rabbits during the hot season [26].

Milk thistle cake can be fermented using probiotic bacteria, such as *Lactobacillus acidophilus*. Fermentation offers multiple benefits, including improved nutrient digestibility and bioavailability through the breakdown of complex fibers and increased content of essential vitamins, such as B vitamins [27]. A review [28] highlights the role of probiotics in enhancing rabbit health, including improvement in gut health and reduction in the abundance of harmful bacteria such as *E. coli* and *Salmonella*, which together contribute to better productive performance.

The bioactive compound silymarin, with its well-documented antioxidant and hepatoprotective properties, may counteract oxidative stress induced by elevated temperatures. Environmental stressors such as heat and noise can compromise physiological homeostasis, reduce feed efficiency, and negatively impact growth and reproductive performance in rabbits. Nutritional interventions that enhance antioxidant defense represent a promising strategy to improve animal resilience under these stress conditions.

Milk thistle, as a natural source rich in silymarin, has attracted attention as a feed additive due to its protective effects. Fermentation of milk thistle seed cake may further increase the bioavailability and biological activity of its flavonolignans, thereby enhancing physiological responses and productivity.

This study investigates the combined effects of environmental stressors and dietary supplementation with milk thistle seed cake—both non-fermented and fermented—on rabbit performance, physiological parameters, and welfare. Specifically, we aim to (1) determine the impact of elevated temperature on growth, feed conversion, and general health across dietary groups; (2) assess whether noise stress alters selected blood parameters and whether milk thistle supplementation mitigates these changes; and (3) evaluate the effects of different diets on carcass yield. We hypothesize that fermented milk thistle will provide the greatest protective effects against heat and noise stress, improving growth, physiological markers, and slaughter yield. The findings can inform sustainable nutritional strategies that mitigate stress effects and support efficient, welfare-oriented rabbit production systems.

## 2. Materials and Methods

All animal procedures were approved by the [Expert Committee for Ensuring the Welfare of Experimental Animals of the Czech University of Life Sciences Prague)] (approval No. [MZE-13122/2022-13114] Approval Date: 11 March 2022) and were conducted in accordance with Act No. 246/1992 Coll., the Czech Act on the Protection of Animals against Cruelty (adopted by the Czech National Council on 15 April 1992; as amended).

### 2.1. Experimental Design

The experimental animals were housed in the demonstration and experimental barn of the Czech University of Life Sciences Prague, Faculty of Agrobiology, Food and Natural Resources, Department of Husbandry and Ethology of Animals. They were kept in metal double-decker fattening cages in a room equipped with automatic air conditioning. Before the start of the experiment, the housing area was disinfected once with an ECA-CID-P aerosol (EcaTech, LTd, Brno, Czech Republic) prior to the placement of the rabbits. Feed and water were provided ad libitum. An anticoccidial solution (Emanox PMX; Biokron Ltd., Blučina, Czech Republic) was added to the drinking water for all rabbits during the first 10 days of the experiment.

Ninety HYLA broiler rabbits were randomly assigned to three groups of 30 animals each. The groups were designated as C (control, fed a standard total mixture for fattening rabbits), SMT (fed the standard mixture with 2% standard non-fermented milk thistle seed cakes), and FMT (fed the standard mixture with 2% fermented mixture using *Lactobacillus acidophilus*, consisting of milk thistle seed cakes and substrate of alfalfa meal and barley meal in a 50:50 ratio).

A habituation period to the experimental feed mixture was conducted from day 35 to day 42 post-weaning. Pellet diets were administered from day 35 until slaughter at 63–77 days of age, depending on the live weight of the rabbits. Nutritional requirements were met throughout the experiment, and the diets were balanced. The nutrient composition of the diets is shown in Table 1.

The health status of each animal was monitored daily, and body weight was recorded weekly throughout the experiment. The experimental diets were offered individually in feeders with a capacity of 1.6 kg. Feed refusals (uneaten feed) were weighed weekly to determine feed intake, and feeders were subsequently refilled to 1.6 kg according to the animals’ group allocation. Body weight and feed refusals were measured using a digital scale (TRONIX BX 10000; TRONIX, Prague, Czech Republic). Average daily feed intake for each rabbit was calculated from weekly individual intake, and group mean intake was determined. Feed conversion ratio (FCR) was calculated as FCR = feed intake/weight gain.

A detailed timeline of the experimental procedures is presented in Table 2.

Chemical analyses of the diets and feces were performed using the AOAC [29] methods for the determination of ash, crude protein (CP), crude fiber (CF), dry matter (DM), and ether extract (EE). Nitrogen-free extract (NFE) was calculated using the following formula: NFE = 1000 – ash (g) – CP (g) – CF (g) – EE (g). Acid detergent lignin (ADL) was analyzed according to a conventional fiber analysis method [30].

Digestibility of nutrients, including ash, crude protein (CP), ether extract (EE), crude fiber (CF), and nitrogen-free extract (NFE), was determined by the indicator method using ADL lignin.

Digestible energy (DE) was determined by formula [31]: DE = (dCP × 0.02385) + (dEE × 0.03778) + (dCF ×  0.01628) + (dNFE × 0.01711); symbol “d” means “digestible” (%) [31].

All complete feed mixtures were based on the control feed mixture (C), which contained alfalfa meal, oat meal, wheat bran, malt sprouts, sunflower seed meal, barley meal, calcium carbonate, monocalcium phosphate, dried whey meal, sodium chloride, and dried apple pulp. In the granulated SMT mixture, 2% standard non-fermented milk thistle seed cakes were added. In the granulated FMT mixture, 2% fermented substrate was added, consisting of 1% milk thistle seed cakes and 1% substrate.

Table 3 shows the composition of the feed mixtures for C, SMT, and FMT, with respect to the selected flavonolignans and their average amounts.

### 2.2. Animal Sampling

Whole-blood samples were obtained from the animals and immediately processed for analysis.

Animal-origin samples were collected at the end of the experiment and stored at −20 °C prior to HPLC analysis using a Konelab™ 20XT clinical chemistry analyzer (Thermo Fisher Scientific Oy, Vantaa, Finland). In the event that any animals died during the experiment, the deceased individuals were examined to determine the cause of death.

### 2.3. Production Parameters

Production parameters included growth performance (average daily gain), feed intake, feed conversion, and carcass yield. The experiment was conducted in the experimental barn of the Czech University of Life Sciences, Prague. The rabbits were slaughtered at weekly intervals once they reached a minimum live weight of 2500 g. These intervals were statistically analyzed for comparisons between groups. Growth performance parameters (live weight, average daily gain), feed intake, feed conversion, and carcass parameters (carcass yield I and II, live weight, carcass weight, and weight of liver, head, and kidneys with fat) were compared between 63 and 77 days of age. Carcass yield I and II were determined based on the following formulas:

$$\begin{array}{c} Carcass\;I=(carcass\;weight+head+heart+lungs+liver+kidney\;with\;fat)\\ Carcass\;II=(carcass\;weight+head+liver+kidney\;with\;fat)\\ Carcass\;yeld\;I=\left[\frac{Carcass\;I}{live\;weight}\right]\times 100\\ Carcass\;yeld\;II=\left[\frac{Carcass\;II}{live\;weight}\right]\times 100 \end{array}$$

The number of rabbits with a minimum live weight of 2500 g when slaughtered is shown in Table 4 according to age at slaughter (63, 70, or 77 days).

### 2.4. Blood Samples

The rabbits were stunned using a captive bolt gun and subsequently slaughtered. A total of thirty rabbits (ten from each of the three groups) were processed, and blood samples were collected via exsanguination into heparinized tubes (Sarstedt, Nümbrecht, Germany). The blood samples were promptly centrifuged at 3200 rpm for 10 min, and the resulting plasma and blood fractions were stored in Eppendorf Safe-Lock tubes (up to 1.5 mL; Eppendorf, Hamburg, Germany) at −20 °C until laboratory analysis.

To assess the impact of milk thistle seed cakes on rabbit health, a statistical analysis of blood biochemical parameters was performed, focusing on liver and kidney function, energy metabolism, and stress indicators. The monitored parameters included crude protein (CP), albumin (ALB), alanine transaminase (ALT), aspartate transaminase (AST), alkaline phosphatase (ALP), gamma-glutamyl transferase (GGT), lactate, lactate dehydrogenase (LD), total cholesterol (CHOL), high-density lipoprotein (HDL), low-density lipoprotein (LDL), triacylglycerols (TAGs), beta-hydroxybutyric acid (BHB), free fatty acids (FFAs), creatine kinase (CK), creatinine (CREA), urea, total antioxidant status (TAS), glucose (GLU), calcium (Ca), and inorganic phosphate (Pi).

### 2.5. Chemicals

Pure, mechanically processed fruits of *Silybum marianum* (Silyfeed^®^Basic) were obtained from IREL, Ltd., Miroslavské Knínice, Czech Republic. Buffer components, L-ascorbic acid, and acetic acid were purchased from Sigma-Aldrich (St. Louis, MO, USA). Methanol, acetonitrile, and β-glucuronidase/arylsulfatase from *Helix pomatia* were obtained from Merck (Darmstadt, Germany). Other chemicals were obtained from Sigma-Aldrich (St. Louis, MO, USA). All solutions were prepared using deionized water from reverse osmosis (Ultrapur, Watrex, Prague, Czech Republic). Nitrogen and helium (purity grade of 99.999 % for both) were obtained from Linde Gas (Prague, Czech Republic).

### 2.6. Extraction of Flavonolignans from Diet and Feces Samples

Feed and feces samples were collected and dried at 60 °C for 24 h. To determine dry matter, these pre-dried samples were further dried at 103 ± 2 °C until a constant weight was achieved. Crude protein was analyzed using the Kjeldahl method on a KjelROC Analyzer (LiquidLine, Furulund, Sweden), employing a factor of 6.25 to convert nitrogen content to nitrogen substances. Crude fiber was determined using an ANKOM Fiber Analyzer (ANKOM Technology, Macedon, NY, USA) by measuring the non-hydrolyzable residue after hydrolysis with acid detergent (H_2_SO_4_) and alkaline detergent (NaOH), with the ash content subtracted from the residue. Crude fat content was analyzed using the Soxhlet method, with at least 10 reflux cycles per hour, using diethyl ether as the extractant. Ash content was determined by ashing the sample at 550 ± 20 °C until a constant weight was achieved. Lignin was measured using an ANKOM device by calculating the mass balance after cellulose and other organic substances were dissolved in an acid detergent. Fiber content was analyzed using 72% H_2_SO_4_ according to ISO 13906 standards [32]. Nitrogen-free extractive (NFE) content was calculated using the following formula: NFE = dry matter (g/kg) − [NS content (g/kg) + fiber content (g/kg) + fat content (g/kg) + ash content (g/kg) [18].

### 2.7. Antioxidant Capacity

Analyses of total antioxidant status (TAS) were performed using the Konelab™ 20XT clinical chemistry analyzer (Thermo Fisher Scientific Oy, Vantaa, Finland). The TAS assay was conducted using the Randox Laboratories Total Antioxidant Status (TAS) kit, which was optimized for use with the Thermo Konelab platform (Randox Laboratories Ltd., Crumlin, UK), in accordance with the manufacturer’s instructions (IFU).

### 2.8. Glutathione Peroxidase (GPx) Activity Assay

GPx activity in erythrocytes was measured spectrophotometrically at a wavelength of 340 nm. The reaction mixture contained 0.39 mM GSH, 0.19 mM NADPH, and 1.55 U/mL glutathione reductase in an assay buffer composed of 50 mM Tris and 0.1 mM EDTA at pH 7.6. A 10 μL erythrocyte sample was added to the mixture, and the enzymatic reaction was initiated with 0.1% cumene hydroperoxide. GPx activity was expressed as μkat/g of hemoglobin [28].

### 2.9. Heat Stress

The ambient temperature in the housing facility was maintained at 17 ± 1 °C throughout the trial, except for the first week of the feeding experiment, during which the temperature was increased to 25 ± 1 °C for 5 days (42–46 days of age). Relative humidity was maintained at approximately 65% throughout the entire experiment.

### 2.10. Noise Stress

Loud or sudden noises are a major cause of stress for rabbits as prey animals with highly sensitive hearing. Five rabbits from each group were exposed to noise stress lasting for 10–20 min at levels ranging from 63.2 to 74.7 dB on the 70th day of age, before slaughter and blood collection.

### 2.11. Statistical Analysis

Data were statistically processed using STATISTICA.CZ, version 10.0 (Czech Republic). Classical one-way analysis of variance (ANOVA) was performed, followed by Scheffé’s test for all parameters, including nutrient digestibility, silymarin content, production parameters, and biochemical blood parameters.

Blood parameters were further subjected to multifactorial analysis using ANOVA with post hoc Scheffé’s test. The variables included individual blood parameters and exposure to the noise stressor. Thermal stress was not considered, as all rabbits were maintained under the same temperature conditions regardless of group. Productive parameters were not evaluated, since short-term noise exposure immediately before slaughter was not expected to affect growth rate, carcass yield, or related production traits.

## 3. Results

### 3.1. Biochemical Blood Indicators

The average values of the 22 monitored blood parameters in half of the rabbits in each group, divided according to their feed mixture and exposure to noise stress, are shown in Table 5. The evaluated parameters included enzymes (e.g., alkaline phosphatase—ALP, aspartate transaminase—AST, glutathione peroxidase—GSH-Px), total protein (TP), urea, glucose (GLU), high-density lipoprotein (HDL), low-density lipoprotein (LDL), creatinine (CREA), free fatty acids (FFAs), calcium (Ca), and inorganic phosphate (Pi).

The blood albumin level in non-stressed rabbits was significantly higher in the FMT group (1% fermented milk thistle + 1% substrate in diet) compared to the control group (C; without supplementation of milk thistle), which corresponded with the total protein content in the blood of the monitored rabbits (Table 5). A statistically significant difference was also observed in the levels of mineral substances (Ca and Pi), with the FMT group showing higher concentrations of these minerals in the blood than the control group (C). Analysis of free fatty acids (FFAs) and triacylglycerols (TAGs) indicated higher fat mobilization in the FMT group. The SMT group (2% non-fermented milk thistle in diet) exhibited a higher level of glutathione peroxidase (GSH-Px), but lower levels of total antioxidant status (TAS) and creatinine (CREA), compared to the control group (C). The highest values of GSH-Px, TAS, and CREA were observed in the FMT group.

The same blood parameters were analyzed in the other half of the rabbits after exposure to noise stress. These results are presented in Table 6.

Regarding blood parameters, among the rabbits exposed to noise stress, the SMT group showed the highest levels of lactate dehydrogenase (LD), lactate, and triacylglycerols (TAGs) compared to the other groups; however, these differences were not statistically significant (*p* > 0.05). A statistically significant difference (*p* < 0.05) was observed in plasma GSH-Px levels, with the FMT group exhibiting nearly four times higher GSH-Px levels than the control group and more than twice as high as the SMT group (Table 5). A similar trend was observed for total antioxidant status (TAS), although the differences were not statistically significant. The FMT group also showed the highest level of free fatty acids (FFAs), while the control group exhibited the lowest levels.

Table 7 presents the mean values of the monitored blood parameters for all rabbits from each group, regardless of stress exposure. Table 8 shows the effects of noise stress on the rabbits, irrespective of their diet.

Statistically significant differences were observed among the groups with different diets with regard to GSH-Px activity as well as levels of FFAs, inorganic phosphorus, urea, and total antioxidant status (TAS) in blood plasma. The FMT group exhibited the highest values (*p* < 0.05) for these parameters compared to the other groups, except for urea, where a statistically significant difference was observed only in comparison with the control group.

Rabbits exposed to noise stress exhibited higher values on more than half of the evaluated blood parameters (Table 8) compared to non-stressed animals. The levels of ALT, AST, BHB, and GGT were comparable between groups, whereas for the other six parameters, higher values were observed in rabbits not exposed to noise stress. The levels of glucose (GLU) and total antioxidant status (TAS) were significantly higher (*p* < 0.05) in the stressed group, while the level of the liver enzyme alkaline phosphatase (ALP) was significantly lower (*p* < 0.05).

Table 9 presents the biochemical parameters according to the different diets (control diet without milk thistle; SMT diet with 2% non-fermented milk thistle; and FMT diet with 1% fermented milk thistle + 1% substrate) and stress factor (exposure to noise stress before slaughter or not). A factorial analysis (diet × noise) was performed, which indicated whether diet or stress exposure had a statistically significant effect based on the sum of squares (SS), mean square (MS), degree of freedom (df), ratio of variances (F), and *p*-value (*p*), indicated whether the diet or stress factor had a statistically significant effect.

The multifactorial analysis of blood parameters revealed statistically significant interactions between diet type and stress exposure on GSH-Px activity and levels of TAS, FFAs, and GLU. Glutathione peroxidase (GSH-Px) activity was higher in the non-stressed FMT group than in the noise-stressed control group (*p* < 0.001) and the stressed SMT group (*p* < 0.01). GSH-Px activity was also higher (*p* < 0.01) in the noise-stressed FMT group compared with the non-stressed control group, the non-stressed SMT group, and both the noise-stressed control and SMT groups. Total antioxidant status (TAS) was lower in the non-stressed SMT group than in the noise-stressed FMT group (*p* < 0.05). Free fatty acids (FFAs) were elevated (*p* < 0.05) in the noise-stressed FMT group compared with the non-stressed control and SMT groups. Glucose (GLU) levels were lower in the non-stressed control group (*p* < 0.05) compared with both the noise-stressed control and SMT groups. No other statistically significant differences were observed (*p* > 0.05).

### 3.2. Production Analysis 

**Figure 1:**
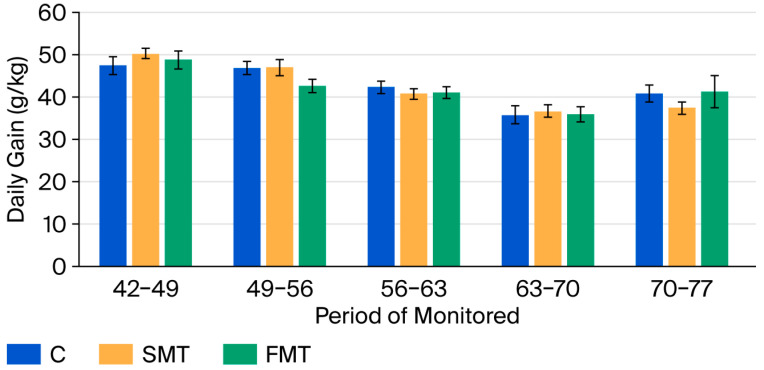
Average daily gain in rabbits during five monitored periods (C, control; SMT, 2% non-fermented milk thistle; FMT, 1% fermented milk thistle + 1% substrate).

Table 10 and Figure 1 show the average daily gain of rabbits during the fattening period from 42 to 77 days, divided into five intervals. During days 42–49 and 49–56, the SMT group exhibited the highest average daily gain, followed by the FMT group, with the C group showing the lowest gain. A similar trend was observed during days 63–70. In contrast, during days 56–63 and 70–77, the SMT group showed the lowest daily gain.

The SMT group exhibited the highest average live weights at all six measured time points. In contrast, the FMT group showed lower average weights after day 56. Detailed results are presented in Table 11.

Feed intake was recorded weekly for all three monitored groups from days 42 to 77 of age. These values were subsequently used to calculate the average weekly feed intake and the average daily intake. The average weekly intake values are presented in Table 12.

The SMT group consistently exhibited the highest weekly feed intake across all time periods (Table 11), whereas the FMT group showed the lowest feed intake throughout the experiment. Statistically significant differences (*p* < 0.05) were observed between these groups from day 49 onwards.

**Figure 2:**
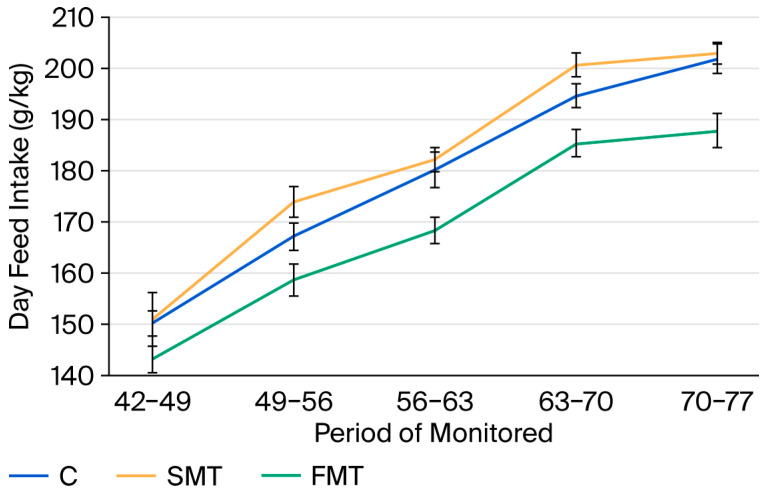
Average daily feed intake across different monitored periods (C, control; SMT, 2% non-fermented milk thistle; FMT, 1% fermented milk thistle + 1% substrate).

These significant differences (*p* < 0.05) were also reflected in the average feed intake across distinct periods, as presented in Table 13 and Figure 2.

Feed conversion, an indicator of the efficiency with which ingested feed is converted into weight gain, was lowest in the C group during days 49–56, 56–63, and 70–77 of the experiment. During days 70–77, the FMT group showed the lowest feed conversion. In most cases, the SMT group exhibited the highest feed conversion. However, these differences were not statistically significant (Table 14).

**Figure 3:**
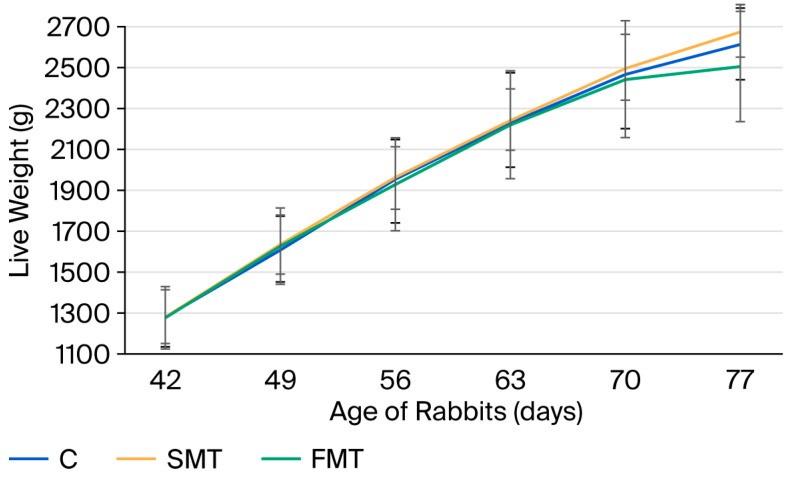
Growth curves of rabbits fed different diets (C, control; SMT, 2% non-fermented milk thistle; FMT, 1% fermented milk thistle + 1% substrate).

The growth curves of the rabbits fed different diets are shown in Figure 3. Until day 63 of age, all three groups exhibited very similar growth performance. After this time point, a slight divergence appeared, with the SMT and C groups showing somewhat higher growth compared to the FMT group. This trend was more pronounced at the final weighing (day 77), although the differences were not statistically significant (*p* > 0.05).

Table 15 shows the average fecal values of ash, crude protein (CP), ether extract (EE, an indicator of fat digestibility), crude fiber (CF), nitrogen-free extract (NFE), and digestible energy (DE). Based on the results for nutrient digestibility, the highest overall digestibility was shown in the C group, except for crude protein, which was highest in the SMT group. The lowest digestibility of the evaluated nutrients was observed in the FMT group. The most pronounced difference was found in the apparent digestibility of ash.

The most pronounced difference was observed in the apparent digestibility of ash. The FMT group showed 18.2% lower ash digestibility compared with the control group. Other notable differences were found in the digestibility of crude fiber (approximately 10.69%), nitrogen-free extract (approximately 7.16%), ether extract (approximately 6.56%), and digestible energy (approximately 0.89 MJ), with the FMT group exhibiting the lowest values and the control group the highest. The highest crude protein digestibility was recorded in the SMT group, while the FMT group showed the lowest value, with a decrease of 8.17%. The inclusion of non-fermented milk thistle (SMT) significantly improved (*p* < 0.05) the digestibility of nitrogenous substances compared with both the C and FMT diets. The ratios of crude protein content in the diet to average daily gain over the five monitored periods are presented in Table 16.

Starting from 49 days of age, the conversion of crude protein to average daily gain was most efficient in the control group, showing significant differences compared with both groups fed the milk thistle-supplemented diets during the periods of 49–56 and 56–63 days (*p* < 0.05). The group fed the FMT diet showed the lowest crude protein-to-gain ratios across almost all observation periods, except for days 63–70, when the lowest ratio was observed in the SMT group.

The results presented in Table 16 show a consistent trend of increasing crude protein conversion with age across all groups. In the FMT group, protein conversion steadily worsened throughout the entire observation period. In contrast, both the C and SMT groups showed some improvement in crude protein conversion during the last monitored period (days 70–77) compared with the previous period (days 63–70). However, the values in this final period did not reach those observed during days 56–63.

### 3.3. Carcass Yield

The evaluated carcass yield parameters for the total observation period are presented in Table 17. Up to 63 days of age, only a few rabbits reached slaughter weight (>2500 g): two individuals in the C group, none in the SMT group, and two in the FMT group. These data are presented in separate tables: carcass yield is presented in Table 17 for 70-day-old rabbits and Table 18 for 77-day-old rabbits. Carcass yield was not determined for seven rabbits in the C group, six in the SMT group, and seven in the FMT group, as they had not reached slaughter weight even at 77 days and were classified as ungrown in Table 2. 

In the control group (C) fed the diet without milk thistle, nearly 7% of rabbits reached slaughter weight by 63 days, the same proportion as in the FMT group (Table 4). By 70 days, nearly half of the FMT group, almost one-third of the C group, and more than one-third of the SMT group had reached slaughter weight. On day 77, the highest number of slaughtered rabbits was recorded in the SMT group (nearly half of the group), followed by 40% in the C group and just under one-quarter in the FMT group.

At 70 days of age, the highest number of slaughtered animals was recorded in the FMT group (16 out of 30), while the lowest was in the SMT group (10 out of 30). Nevertheless, carcass weight, Carcas I, and Carcas II during this period (Table 18) were highest in C and lowest in SMT (*p* < 0.05).

In the following week, however, a reversal occurred: in the FMT, only half as many animals were slaughtered compared to the previous week. Of all individuals slaughtered at 77 days of age, 42% belonged to the SMT. At the same time, SMT showed the highest values in almost all selected carcass yield parameters (Table 19).

For liver and kidney weights, the overall differences between groups were minimal (Table 17). More noticeable variations appeared across individual monitoring intervals. At 70 days of age, the highest liver and kidney weights were recorded in the control group (C), followed by the FMT group, and the lowest in the SMT group (Table 18). At 77 days, the trend was reversed, with the highest weights recorded in the SMT group and the lowest in the C group (Table 19). However, these differences were not significant.

## 4. Discussion

Silymarin, contained in milk thistle, exhibits several biological activities, including anti-inflammatory, antioxidant, immunomodulatory, and hepatoprotective effects [33]. These effects can be reflected in blood parameters. According to the authors of [34], blood samples provide vital information on physiological status, and the levels of blood metabolites recorded in our study are consistent with the findings reported by other researchers [35]. Dietary supplementation with silymarin has been shown to positively influence blood glucose, urea, creatinine, and reactive oxygen species (ROS), which are byproducts of aerobic metabolism.

According to [33], intraperitoneal administration of silybin accelerates ribosome formation, thereby enhancing protein synthesis. Other antioxidant mechanisms of silymarin include the inhibition of ROS-producing enzymes and the stimulation of protective molecules such as heat shock proteins. In our experiment, glutathione peroxidase (GSH-Px) activity was higher in both non-stressed and noise-stressed rabbits fed a diet supplemented with 1% fermented milk thistle and 1% substrate (FMT) compared to rabbits fed the control (C) and 2% non-fermented milk thistle (SMT) diets.

Analysis of blood parameters further revealed that the interaction between diet and stress exposure affected antioxidant and metabolic markers. In particular, the total antioxidant status (TAS) and free fatty acid (FFA) levels were elevated in noise-stressed FMT-fed rabbits, reflecting increased lipid mobilization. Glucose (GLU) concentration was lower in non-stressed control rabbits than in stressed animals, indicating stress-related metabolic adjustments. Collectively, these findings suggest that fermented milk thistle supplementation strengthens antioxidant defense and modulates energy metabolism under stress conditions, complementing the observed increases in GSH-Px, total protein, and albumin.

The crude protein concentration in plasma was higher in both the SMT and FMT groups than in the control group, supporting previous reports of enhanced protein synthesis [33]. In stressed rabbits fed the control diet, urea, cholesterol, and triacylglycerol (TAG) levels were elevated compared with non-stressed control animals, whereas in the noise-stressed FMT group, albumin, total protein, glucose, urea, creatinine, cholesterol, and TAG levels were comparatively maintained, indicating that milk thistle supplementation mitigated the negative effects of stress [22,24,32]. Both the SMT and FMT groups appeared to have greater protein availability for anabolic processes, including muscle fiber synthesis, compared to the non-stressed controls.

These results are consistent with previous studies reporting that stress alters blood metabolites by affecting energy and protein metabolism [2,36,37]. Noise stress negatively impacts the auditory, nervous, endocrine, and cardiovascular systems [38], yet FMT supplementation was found to support antioxidant defenses and preserve protein and energy balance. Our findings align with other studies on oxidative stress responses, where enhanced GSH-Px activity was observed in animals supplemented with silymarin under stress conditions [39].

The authors of [40] found that heat stress decreases crude protein and calcium in the blood of growing rabbits while increasing glucose and cholesterol levels. Elevated creatinine levels have also been associated with possible muscle damage [41]. A previous study [42] showed that heat-stressed rabbits exhibit stronger physiological stress responses than noise-exposed rabbits, although the latter display increased creatine kinase levels, indicating muscular strain. In our experiment, noise-stressed rabbits exhibited increased levels of total protein, glucose, creatine kinase, and creatinine, but these changes were less pronounced than those reported by [40], indicating a milder effect of noise stress.

A study [43] on New Zealand White rabbits demonstrated that heat stress reduces final body weight and weight gain while increasing the feed conversion ratio. In our study, stressed rabbits exhibited decreased crude protein but elevated creatinine, urea, and aspartate transaminase levels, as well as reduced GSH-Px activity.

It can be concluded that long-term exposure to elevated ambient temperatures induces oxidative and heat stress, impairing physiological functions. In our experiment, the group receiving 2% milk thistle achieved the highest average daily gain, confirming its positive effect on growth during the early fattening phase. This finding is consistent with our previous study [44], in which rats fed milk thistle-enriched diets (10–20%) showed increased daily weight gain compared with controls.

Across four of the five monitored periods, rabbits fed milk thistle exhibited the highest average daily gain, consistent with the findings of [45]. That study compared 1% unfermented and 0.5% fermented milk thistle and reported comparable performance between the control and 1% groups, while the 0.5% group showed lower gain. Similarly, in our experiment, the 1% FMT group had the lowest daily weight gain, though the differences were not statistically significant (*p* > 0.05).

The authors of [45] also observed the highest feed intake in rabbits receiving 0.5% fermented milk thistle and the lowest in those receiving 1% unfermented milk thistle. We found a similar pattern, with the highest feed intake observed in the 2% SMT group and the lowest in the 1% FMT group, showing statistically significant differences. While [45] found minimal differences in feed conversion ratios, our results showed greater variability across treatments, although the differences were not statistically significant (*p* > 0.05). The SMT group showed a statistically higher total feed intake than the FMT group.

In [45], the highest slaughter weight was recorded in the 0.5% FMT group, whereas the lowest was observed in the 1% SMT group. In contrast, in our experiment, fermented milk thistle did not result in superior slaughter or carcass weight.

Similarly, Ref. [46] reported that *Silybum marianum* supplementation improved growth, feed conversion, and immune function in quails, showing increased feed intake, body weight, and relative liver weight. These results partially correspond with our findings, except that improved feed conversion efficiency was not confirmed. Digestibility of selected nutrients was reduced in the milk thistle-supplemented groups, particularly in the FMT group. Comparable improvements in growth parameters—such as weight gain and specific growth rate—were also reported in fish species fed *Silybum marianum* extract [46,47].

## 5. Conclusions

Dietary supplementation with milk thistle, in both fermented and non-fermented forms, demonstrated beneficial effects on the antioxidant status and metabolic balance of rabbits, particularly under non-stress conditions. Fermented milk thistle enhanced antioxidant enzyme activity and mineral homeostasis, while non-fermented milk thistle improved growth and carcass yield in later fattening stages. Although no consistent effects on production parameters were observed across treatments, milk thistle supplementation contributed to more stable physiological responses and appeared to mitigate the impact of environmental stress. These findings suggest that the inclusion of milk thistle in rabbit diets represents a promising natural strategy to support health, welfare, and sustainable productivity under varying environmental conditions.

## Figures and Tables

**Table 1:** Chemical composition of the experimental diets.

	C	SMT	FMT
Dry matter (g/kg)	1000	1000	1000
Ash (g/kg)	90.44	92.13	89.76
Crude protein (g/kg)	151.97	182.40	182.92
Ether extract (g/kg)	29.96	36.19	34.57
Crude fibre (g/kg)	216.44	216.31	216.17
ADL-lignin (g/kg)	56.31	53.86	56.95
NFE (g/kg)	511.18	472.96	476.57
Calcium (g/kg)	12	12	12
Phosphorus (g/kg)	6	6	6
Natrium (g/kg)	4	4	4
Vitamin A (IU/kg)	11,500	11,500	11,500
Vitamin D_3_ (IU/kg)	1400	1400	1400
Ferrum (mg/kg) Source: FeSO_4_·H_2_O	30	30	30
Iodine (mg/kg) Source: Ca(IO_3_)_2_	1	1	1
Copper (mg/kg) Source: CuSO_4_·5H_2_O	14	14	14
Manganese (mg/kg) Source: MnO	35	35	35
Zinc (mg/kg) Source: ZnO	35	35	35
Selenium (mg/kg) Source: Na_2_SeO_3_	0.1	0.1	0.1

C, control; SMT, 2% non-fermented milk thistle; FMT, 1% fermented milk thistle + 1% substrate.

**Table 2:** Timeline of experimental procedures in relation to the age of rabbits.

Age of Rabbits		C	SMT	FMT
35–41 days	procedure	habituation		
	n	30	30	30
42 days	procedure	individual weighing of weekly weight gains and feed refusals		
	n	30	30	30
42–46 days	procedure	temperature stress		
	n	30	30	30
49 days	procedure	individual weighing of weekly weight gains and feed refusals		
	n	30	30	30
56 days	procedure	individual weighing of weekly weight gains and feed refusals		
	n	30	30	30
63 days	procedure	individual weighing of weekly weight gains and feed refusals		
	n	30	30	30
	procedure	slaughter without noise stress		
	n	2	0	2
70 days	procedure	individual weighing of weekly weight gains and feed refusals		
	n	28	30	28
	procedure	noise stress before slaughter		
	n	5	5	5
	procedure	slaughter without noise stress		
	n	5	5	9
77 days	procedure	individual weighing of weekly weight gains and feed refusals		
	n	18	20	14
	procedure	slaughter without noise stress		
	n	11	14	7

n = number of individuals; C, control; SMT, 2% non-fermented milk thistle; FMT, 1% fermented milk thistle + 1% substrate.

**Table 3:** Silymarin content (g/kg) and its main flavonolignans in the experimental diets.

(g/kg)	C	SMT Average ± SE	FMT Average ± SE
Total silymarin	0	1.450 ± 0.000	0.710 ± 0.000
Silybinin A	0	0.545 ± 0.000	0.333 ± 0.008
Silybinin B	0	0.449 ± 0.015	0.349 ± 0.015
Isosilybinin A	0	0.158 ± 0.077	0.098 ± 0.001
Isosilybinin B	0	0.044 ± 0.000	0.033 ± 0.000
Silychristin	0	0.502 ± 0.015	0.301 ± 0.008
Silydianin	0	0.044 ± 0.000	0.033 ± 0.000

SE = standard error; C, control; SMT, 2% non-fermented milk thistle; FMT, 1% fermented milk thistle + 1% substrate.

**Table 4:** General parameters of animals with a minimum live weight of 2500 g according to age at slaughter.

	C	SMT	FMT
Age at slaughter: 63 days	2	-	2
Age at slaughter: 70 days	10	10	14
Age at slaughter: 77 days	11	14	7
Ungrown	7	6	7
Death	0	0	0
Total number	30	30	30

C, control; SMT, 2% non-fermented milk thistle; FMT, 1% fermented milk thistle + 1% substrate.

**Table 5:** Biochemical blood parameters in non-stressed rabbits according to their feed mixture.

	C Average ± SE	SMT Average ± SE	FMT Average ± SE
Number of animals	5	5	5
Albumin (g/L)	28.18 ± 1.29 ^b^	30.48 ± 1.19 ^ab^	35.35 ± 1.38 ^a^
ALP (µkat/L)	2.46 ± 0.29	3.02 ± 0.28	3.04 ± 0.40
ALT (µkat/L)	0.53 ± 0.04	0.60 ± 0.04	0.55 ± 0.07
AST (µkat/L)	0.24 ± 0.03	0.19 ± 0.02	0.20 ± 0,01
BHB (mmol/L)	0.05 ± 0.02	0.03 ± 0.01	0.07 ± 0.00
Ca (mmol/L)	2.63 ± 0.10 ^b^	2.85 ± 0.08 ^b^	3.37 ± 0.14 ^a^
CHOL (mmol/L)	1.42 ± 0.25	1.36 ± 0.17	1.61 ± 0.18
HDL (mmol/L)	0.53 ± 0.06	0.44 ± 0.05	0.59 ± 0.05
LDL (mmol/L)	0.89 ± 0.19	0.92 ± 0.14	1.03 ± 0.14
CK (µkat/L)	21.06 ± 3.59	18.74 ± 3.46	24.17 ± 2.13
CREA (µmol/L)	60.64 ± 8.40 ^ab^	48.37 ± 1.12 ^b^	72.37 ± 4.94 ^a^
GGT (µkat/L)	0.09 ± 0.01	0.13 ± 0.02	0.13 ± 0.02
GLU (mmol/L)	5.55 ± 0.19 ^b^	6.14 ± 0.29 ^b^	7.48 ± 0.40 ^a^
GSH-Px (µkat/L)	488.68 ± 49.44 ^b^	630.42 ± 47.52 ^b^	1480.40 ± 190.40 ^a^
LD (µkat/L)	2.66 ± 0.23	2.62 ± 0.38	3.06 ± 0.26
Lactate (mmol/L)	2.36 ± 0.23	2.54 ± 0.26	3.96 ± 0.77
FFA (mmol/L)	0.10 ± 0.03 ^b^	0.10 ± 0.01 ^b^	0.26 ± 0.05 ^a^
Pi (mmol/L)	1.53 ± 0.09 ^b^	1.54 ± 0.05 ^b^	2.24 ± 0.13 ^a^
TP (g/L)	42.98 ± 2.48 ^b^	44.51 ± 1.75 ^b^	54.25 ± 1.87 ^a^
TAS (mmol/L)	0.79 ± 0.02 ^ab^	0.75 ± 0.13 ^b^	0.95 ± 0.04 ^a^
TAG (mmol/L)	0.84 ± 0.15	1.13 ± 0.16	1.28 ± 0.14
Urea (mmol/L)	5.48 ± 0.29 ^ab^	5.44 ± 0.41 ^b^	7.56 ± 0.77 ^a^

SE = standard error; means within the same row marked with different letters (a,b) differ significantly (*p* < 0.05); C, control; SMT, 2% non-fermented milk thistle; FMT, 1% fermented milk thistle + 1% substrate.

**Table 6:** Biochemical parameters in stressed rabbits in the three experimental diet groups.

	C Average ± SE	SMT Average ± SE	FMT Average ± SE
Number of animals	5	5	5
Albumin (g/L)	33.69 ± 0.45	33.02 ± 0.64	32.60 ± 2.30
ALP (µkat/L)	2.32 ± 0.15	2.67 ± 0.08	2.21 ± 0.16
ALT (µkat/L)	0.64 ± 0.02	0.56 ± 0.12	0.49 ± 0.04
AST (µkat/L)	0.21 ± 0.02	0.24 ± 0.02	0.21 ± 0.01
BHB (mmol/L)	0.04 ± 0.01	0.06 ± 0.01	0.03 ± 0.01
Ca (mmol/L)	3.06 ± 0.08	3.10 ± 0.06	3.06 ± 0.19
CHOL (mmol/L)	1.48 ± 0.17	1.18 ± 0.09	1.14 ± 0.14
HDL (mmol/L)	0.61 ± 0.07	0.45 ± 0.06	0.53 ± 0.06
LDL (mmol/L)	0.88 ± 0.12	0.73 ± 0.10	0.60 ± 0.09
CK (µkat/L)	21.40 ± 2.07	30.71 ± 3.94	20.38 ± 2.03
CREA (µmol/L)	56.90 ± 1.02	62.54 ± 10.80	64.17 ± 3.42
GGT (µkat/L)	0.11 ± 0.01	0.12 ± 0.01	0.13 ± 0.01
GLU (mmol/L)	7.42 ± 0.35	7.27 ± 0.26	6.87 ± 0.39
GSH-Px (µkat/L)	395.34 ± 46.84 ^b^	691.80 ± 41.84 ^b^	1534.94 ± 149.25 ^a^
LD (µkat/L)	2.21 ± 0.27	3.24 ± 0.38	2.43 ± 0.20
Lactate (mmol/L)	3.19 ± 0.46	3.79 ± 0.98	3.03 ± 0.39
FFA (mmol/L)	0.11 ± 0.01 ^b^	0.13 ± 0.02 ^b^	0.32 ± 0.08 ^a^
P_i_ (mmol/L)	1.93 ± 0.13	1.77 ± 0.08	2.07 ± 0.16
TP (g/L)	50.58 ± 0.81	50.47 ± 1.37	50.65 ± 4.03
TAS (mmol/L)	0.86 ± 0.05	0.89 ± 0.03	0.97 ± 0.04
TAG (mmol/L)	1.07 ± 0.13	1.32 ± 0.30	0.90 ± 0.13
Urea (mmol/L)	5.74 ± 0.21	6.09 ± 0.37	6.16 ± 0.24

SE = standard error; a, b means within the same row marked with different letters (a,b) differ significantly (*p* < 0.05); C, control; SMT, 2% non-fermented milk thistle; FMT, 1% fermented milk thistle + 1% substrate.

**Table 7:** Biochemical blood parameters in all rabbits (without considering exposure to noise stress).

	C Average ± SE	SMT Average ± SE	FMT Average ± SE
Number of animals	10	10	10
Albumin (g/L)	30.94 ± 1.12	31.75 ± 0.77	33.98 ± 1.34
ALP (µkat/L)	2.39 ± 0.15	2.84 ± 0.15	2.63 ± 0.24
ALT (µkat/L)	0.58 ± 0.03	0.58 ± 0.06	0.52 ± 0.04
AST (µkat/L)	0.22 ± 0.02	0.22 ± 0.01	0.20 ± 0.01
BHB (mmol/L)	0.04 ± 0.01	0.04 ± 0.01	0.05 ± 0.01
Ca (mmol/L)	2.85 ± 0.09	2.98 ± 0.07	3.22 ± 0.12
CHOL (mmol/L)	1.45 ± 0.14	1.27 ± 0.10	1.38 ± 0.14
HDL (mmol/L)	0.57 ± 0.05	0.45 ± 0.03	0.56 ± 0.04
LDL (mmol/L)	0.88 ± 0.11	0.82 ± 0.09	0.81 ± 0.11
CK (µkat/L)	24.23 ± 2.17	24.73 ± 3.18	22.27 ± 1.52
CREA (µmol/L)	58.77 ± 4.04	55.46 ± 5.64	68.27 ± 3.15
GGT (µkat/L)	0.10 ± 0.01	0.12 ± 0.01	0.13 ± 0.01
GLU (mmol/L)	6.49 ± 0.36	6.70 ± 0.26	7.17 ± 0.28
GSH-Px (µkat/L)	442.01 ± 35.68 ^b^	661.11 ± 31.55 ^b^	1507.67 ± 114.61 ^a^
LD (µkat/L)	2.44 ± 0.18	2.93 ± 0.27	2.75 ± 0.19
Lactate (mmol/L)	2.77 ± 0.28	3.17 ± 0.52	3.50 ± 0.43
FFA (mmol/L)	0.11 ± 0.01 ^b^	0.12 ± 0.02 ^b^	0.29 ± 0.05 ^a^
Pi (mmol/L)	1.73 ± 0.10 ^b^	1.66 ± 0.06 ^b^	2.16 ± 0.10 ^a^
TP (g/L)	46.78 ± 1.77	47.49 ± 1.44	52.45 ± 2.18
TAS (mmol/L)	0.82 ± 0.03 ^b^	0.82 ± 0.04 ^b^	0.96 ± 0.03 ^a^
TAG (mmol/L)	0.95 ± 0.08	1.22 ± 0.16	1.09 ± 0.11
Urea (mmol/L)	5.61 ± 0.17 ^b^	5.76 ± 0.28 ^ab^	6.86 ± 0.44 ^a^

SE = standard error; means within the same row marked with different letters (a,b) differ significantly (*p* < 0.05); C, control; SMT, 2% non-fermented milk thistle; FMT, 1% fermented milk thistle + 1% substrate.

**Table 8:** Biochemical parameters in stressed and non-stressed rabbits without considering the feeding ration.

	Standard Conditions Average ± SE	Noise Stress Average ± SE
Number of animals	15	15
Albumin (g/L)	31.33 ± 1.05	33.11 ± 0.76
ALP (µkat/L)	2.84 ± 0.19 ^a^	2.40 ± 0.09 ^b^
ALT (µkat/L)	0.56 ± 0.03	0.56 ± 0.04
AST (µkat/L)	0.21 ± 0.01	0.21 ± 0.01
BHB (mmol/L)	0.05 ± 0.01	0.04 ± 0.01
Ca (mmol/L)	2.95 ± 0.10	3.08 ± 0.07
CHOL (mmol/L)	1.46 ± 0.11	1.27 ± 0.08
HDL (mmol/L)	0.52 ± 0.03	0.53 ± 0.04
LDL (mol/L)	0.95 ± 0.09	0.73 ± 0.06
CK (µkat/L)	23.33 ± 1.91	24.16 ± 1.96
CREA (µmol/L)	60.46 ± 4.00	61.20 ± 3.61
GGT (µkat/L)	0.12 ± 0.01	0.12 ± 0.01
GLU (mmol/L)	6.39 ± 0.27 ^b^	7.18 ± 0.19 ^a^
GSH-Px (µkat/L)	866.50 ± 132.74	874.03 ± 138.36
LD (µkat/L)	2.78 ± 0.17	2.63 ± 0.20
Lactate (mmol/L)	2.95 ± 0.32	3.34 ± 0.36
FFA (mmol/L)	0.16 ± 0.03	0.19 ± 0.03
Pi (mmol/L)	1.77 ± 0.10	1.93 ± 0.08
TP (g/L)	47.25 ± 1.73	50.56 ± 1.34
TAS (mmol/L)	0.83 ± 0.03 ^b^	0.91 ± 0.02 ^a^
TAG (mmol/L)	1.08 ± 0.08	1.10 ± 0.12
Urea (mmol/L)	6.16 ± 0.39	6.00 ± 0.16

SE = standard error; means within the same row marked with different letters (a,b) differ significantly (*p* < 0.05).

**Table 9:** Univariate tests of significance of biochemical blood parameters in the experimental rabbits.

		SS	MS	df	F	*p*
ALB	Diet	49.54	24.77	2	2.739	0.0848
	Stressor	23.58	23.58	1	2.607	0.1195
	Combination	87.50	43.75	2	4.838	0.0172
ALP	Diet	1.05	0.52	2	1.684	0.2068
	Stressor	1.44	1.44	1	4.638	0.0415
	Combination	0.62	0.31	2	0.992	0.3854
ALT	Diet	0.02	0.01	2	0.582	0.5663
	Stressor	0.00	0.00	1	0.007	0.9360
	Combination	0.05	0.02	2	1.261	0.3015
AST	Diet	0.00	0.00	2	0.440	0.6495
	Stressor	0.00	0.00	1	0.297	0.5909
	Combination	0.00	0.00	2	2.231	0.1292
BHB	Diet	0.00	0.00	2	0.291	0.7503
	Stressor	0.00	0.00	1	0.602	0.4454
	Combination	0.00	0.00	2	5.371	0.0118
Ca	Diet	0.71	0.35	2	5.145	0.0138
	Stressor	0.12	0.12	1	1.692	0.2057
	Combination	0.74	0.37	2	5.454	0.0112
CHOL	Diet	0.16	0.08	2	0.537	0.5914
	Stressor	0.30	0.30	1	1.942	0.1762
	Combination	0.37	0.18	2	1.208	0.3163
HDL	Diet	0.09	0.05	2	2.884	0.0754
	Stressor	0.00	0.00	1	0.074	0.7883
	Combination	0.02	0.01	2	0.696	0.5082
LDL	Diet	0.03	0.01	2	0.151	0.8609
	Stressor	0.34	0.34	1	3.634	0.0687
	Combination	0.21	0.10	2	1.132	0.3389
CK	Diet	33.65	16.83	2	0.379	0.6888
	Stressor	5.26	5.26	1	0.118	0.7338
	Combination	468.72	234.36	2	5.274	0.0126
CREA	Diet	884.7	442.4	2	2.353	0.1167
	Stressor	4.1	4.1	1	0.022	0.8835
	Combination	700.4	350.2	2	1.863	0.1770
GGT	Diet	0.00	0.00	2	3.833	0.0359
	Stressor	0.00	0.00	1	0.000	0.9944
	Combination	0.00	0.00	2	0.579	0.5684
GLU	Diet	2.47	1.24	2	2.374	0.1146
	Stressor	4.72	4.72	1	9.065	0.0061
	Combination	8.10	1.05	2	7.782	0.0025
GSH-Px	Diet	6,334,336	3,167,168	2	56.399	0.0000
	Stressor	425	425	1	0.008	0.931
	Combination	38213	19106	2	0.340	0.715
LD	Diet	1.22	0.61	2	1.408	0.2641
	Stressor	0.18	0.18	1	0.414	0.5259
	Combination	2.29	1.14	2	2.637	0.0922
Lactate	Diet	2.63	1.31	2	0.780	0.4696
	Stressor	1.11	1.11	1	0.655	0.4263
	Combination	6.64	3.32	2	1.968	0.1617
FFA	Diet	0.21	0.11	2	12.642	0.0002
	Stressor	0.00	0.00	1	0.839	0.3687
	Combination	0.00	0.00	2	0.146	0.8651
Pi	Diet	1.46	0.73	2	11.338	0.0003
	Stressor	0.18	0.18	1	2.764	0.1094
	Combination	0.43	0.22	2	3.342	0.0524
TP	Diet	190.81	95.40	2	3.640	0.0416
	Stressor	82.46	82.46	1	3.146	0.0888
	Combination	183.00	91.50	2	3.491	0.0467
TAS	Diet	0.13	0.06	2	7.387	0.0032
	Stressor	0.05	0.05	1	5.264	0.0308
	Combination	0.02	0.01	2	1.074	0.3576
TAG	Diet	0.36	0.18	2	1.254	0.3035
	Stressor	0.00	0.00	1	0.008	0.9295
	Combination	0.58	0.29	2	2.032	0.1531
UREA	Diet	9.30	4.65	2	5.179	0.0135
	Stressor	0.21	0.21	1	0.228	0.6371
	Combination	5.87	2.93	2	3.268	0.0556

SS = sum of squares; MS = mean square; df = degrees of freedom; F = ratio of variances; *p* = *p*-value.

**Table 10:** Average daily gain in rabbits.

Period	C (g/kg)		SMT (g/kg)		FMT (g/kg)	
	n	Average ± SE	n	Average ± SE	n	Average ± SE
42–49	30	47.45 ± 2.12	30	50.33 ± 1.23	30	48.78 ± 2.04
49–56	30	46.89 ± 2.41	30	47.00 ± 1.92	30	42.70 ± 1.57
56–63	30	42.35 ± 1.48	30	40.81 ± 1.20	30	41.12 ± 1.37
63–70	28	35.77 ± 2.13	30	36.71 ± 1.39	28	36.02 ± 1.78
70–77	18	40.83 ± 2.00	20	37.50 ± 1.42	14	41.33 ± 3.75

SE = standard error; n = number of individuals; C, control; SMT, 2% non-fermented milk thistle; FMT, 1% fermented milk thistle + 1% substrate.

**Table 11:** Live weight of rabbits from days 42 to 77 of age.

Period	C (g/kg)		SMT (g/kg)		FMT (g/kg)	
	n	Average ± SE	n	Average ± SE	n	Average ± SE
42 days	30	1265.00 ± 25.56	30	1282.33 ± 24.22	30	1268.57 ± 29.41
49 days	30	1597.14 ± 28.82	30	1634.67 ± 26.64	30	1610.00 ± 34.54
56 days	30	1925.36 ± 36.06	30	1963.67 ± 28.41	30	1908.93 ± 41.56
63 days	30	2221.79 ± 40.39	30	2249.33 ± 27.78	30	2196.79 ± 46.82
70 days	28	2472.14 ± 49.90	30	2506.33 ± 29.71	28	2448.93 ± 54.39
77 days	18	2620.00 ± 40.26	20	2685.50 ± 28.77	14	2512.14 ± 72.42

SE = standard error; n = number of individuals; C, control; SMT, 2% non-fermented milk thistle; FMT, 1% fermented milk thistle + 1% substrate.

**Table 12:** Weekly feed intake of control group and two groups fed fermented or non-fermented form of milk thistle as supplementation across distinct monitored periods.

Period	C (g/kg/Week)		SMT (g/kg/Week)		FMT (g/kg/Week)	
	n	Average ± SE	n	Average ± SE	n	Average ± SE
42–49	30	1051.43 ± 17.55	30	1056.17 ± 36.51	30	1002.50 ± 17.78
49–56	30	1170.00 ± 18.88 ^ab^	30	1217.67 ± 20.47 ^a^	30	1111.07 ± 21.87 ^b^
56–63	30	1261.79 ± 24.42 ^a^	30	1275.67 ± 16.76 ^a^	30	1178.21 ± 18.20 ^b^
63–70	28	1362.86 ± 16.82 ^ab^	30	1405.00 ± 16.07 ^a^	28	1297.68 ± 18.54 ^b^
70–77	18	1413.16 ±20.13 ^a^	20	1420.00 ± 14.83 ^a^	14	1315.00 ± 23.27 ^b^

SE = standard error; n = number of individuals; means within the same row marked with different letters (a,b) differ significantly (*p* < 0.05); C, control; SMT, 2% non-fermented milk thistle; FMT, 1% fermented milk thistle + 1% substrate.

**Table 13:** Average daily feed intake in the three groups of rabbits.

Period	C (g/kg)		SMT (g/kg)		FMT (g/kg)	
	n	Average ± SE	n	Average ± SE	n	Average ± SE
42–49	30	150.20 ± 2.51	30	150.88 ± 5.22	30	143.21 ± 2.54
49–56	30	167.14 ± 2.70 ^ab^	30	173.95 ± 2.92 ^a^	30	158.72 ± 3.12 ^b^
56–63	30	180.26 ± 3.49 ^a^	30	182.24 ± 2.39 ^a^	30	168.32 ± 2.60 ^b^
63–70	28	194.69 ± 2.40 ^ab^	30	200.71 ± 2.30 ^a^	28	185.38 ± 2.65 ^b^
70–77	18	201.89 ± 2.88 ^a^	20	202.86 ± 2.12 ^a^	14	187.86 ± 3.32 ^b^

SE = standard error; means within the same row marked with different letters (a,b) differ significantly (*p* < 0.05); C, control; SMT, 2% non-fermented milk thistle; FMT, 1% fermented milk thistle + 1% substrate.

**Table 14:** Feed conversion of rabbits fed different diets during the experiment.

	C (g/kg)		SMT (g/kg)		FMT (g/kg)	
	n	Average ± SE	n	Average ± SE	n	Average ± SE
49–56	30	3.88 ± 0.10	30	4.07 ± 0.30	30	3.97 ± 0.18
56–63	30	4.56 ± 0.19	30	4.60 ± 0.24	30	4.59 ± 0.25
63–70	28	6.81 ± 0.92	30	6.51 ± 1.11	28	5.75 ± 0.38
70–77	18	5.21 ± 0.32	20	5.79 ± 0.40	14	7.40 ± 2.89

SE = standard error; n = number of individuals; C, control; SMT, 2% non-fermented milk thistle; FMT, 1% fermented milk thistle + 1% substrate.

**Table 15:** The apparent digestibility of nutrients (%) and digestible energy (DE; MJ) from dry matter diets and feces of the three experimental groups.

	C Average ± SE	SMT Average ± SE	FMT Average ± SE
Ash (%)	51.07 ± 0.51 ^a^	49.76 ± 1.35 ^a^	32.87 ± 0.58 ^b^
CP (%)	75.47 ± 0.20 ^b^	78.25 ± 0.40 ^a^	70.08 ± 0.61 ^c^
EE (%)	83.71 ± 0.43 ^a^	82.69 ± 0.47 ^a^	77.15 ± 1.22 ^b^
CF (%)	21.62 ± 1.09 ^a^	20.45 ± 1.99 ^a^	10.93 ± 1.09 ^b^
NFE (%)	72.53 ± 0.22 ^a^	68.76 ± 0.77 ^b^	65.37 ± 0.46 ^c^
DE (MJ)	12.58 ± 0.03 ^a^	12.51 ± 0.11 ^a^	11.69 ± 0.07 ^b^

SE = standard error; means within the same row marked with different letters (a,b,c) differ significantly (*p* < 0.05); C, control; SMT, 2% non-fermented milk thistle; FMT, 1% fermented milk thistle + 1% substrate; CP, crude protein; EE, ether extract; CF, crude fiber; NFE, nitrogen-free extract; DE, digestible energy.

**Table 16:** The ratio of crude protein intake to average daily gain observed in rabbits fed the three experimental diets.

	C		SMT		FMT	
	n	Average ± SE	n	Average ± SE	n	Average ± SE
49–56	30	0.59 ± 0.02 ^b^	30	0.74 ± 0.05 ^a^	30	0.73 ± 0.03 ^ab^
56–63	30	0.69 ± 0.03 ^b^	30	0.84 ± 0.04 ^a^	30	0.84 ± 0.05 ^ab^
63–70	28	1.04 ± 0.14	30	1.19 ± 0.20	28	1.05 ± 0.07
70–77	18	0.79 ± 0.05	20	1.05 ± 0.07	14	1.35 ± 0.53

SE = standard error; n = number of individuals; means within the same row marked with different letters (a,b) differ significantly (*p* < 0.05); C, control; SMT, 2% non-fermented milk thistle; FMT, 1% fermented milk thistle + 1% substrate.

**Table 17:** Carcass yield over the total observation time without considering age at slaughter.

	C	SMT	FMT
	Average ± SE	Average ± SE	Average ± SE
Number of animals	23	24	23
Live weight (g)	2726.21 ± 20.63	2735.67 ± 88.77	2690.42 ± 85.69
Carcass weight (g)	1372.97 ± 13.52	1360.13 ± 16.83	1360.92 ± 13.70
Liver (g)	112.62 ± 2.99	111.00 ± 2.11	110.29 ± 2.52
Head (g)	124.76 ± 1.04	123.17 ± 1.09	124.04 ± 1.25
Kidney with fat (g)	35.52 ± 1.25	36.53 ± 0.90	35.96 ± 1.02
Carcass I (g)	1565.69 ± 14.98	1551.13 ± 17.99	1551.04 ± 14.70
Carcass II (g)	1645.86 ± 16.31	1630.83 ± 18.70	1631.21 ± 15.83
Carcass yield I (%)	61.60 ± 0.57	60.73 ± 0.48	61.74 ± 0.40
Carcass yield II (%)	60.41 ± 0.56	59.58 ± 0.48	60.62 ± 0.40

SE = standard error; C, control; SMT, 2% non-fermented milk thistle; FMT, 1% fermented milk thistle + 1% substrate.

**Table 18:** Carcass yield at 70 days of age.

	C	SMT	FMT
	Average ± SE	Average ± SE	Average ± SE
Number of animals	10	10	14
Live weight (g)	2763.33 ± 53.33	2673.00 ± 33.60	2675.00 ± 18.48
Carcass weight (g)	1404.44 ± 27.06 ^a^	1289.10 ± 35.50 ^b^	1363.79 ± 15.26 ^ab^
Liver (g)	112.67 ± 8.85	102.20 ± 2.95	106.93 ± 3.46
Head (g)	126.33 ± 1.31	121.00 ± 2.72	124.71 ± 1.97
Kidney with fat (g)	37.00 ± 2.79	32.90 ± 1.35	34.64 ± 1.30
Carcass I (g)	1603.22 ± 29.64 ^a^	1473.80 ± 37.62 ^b^	1553.79 ± 16.75 ^ab^
Carcass II (g)	1680.44 ± 36.63 ^a^	1545.20 ± 37.74 ^b^	1630.07 ± 18.26 ^ab^
Carcass yield I (%)	62.22 ± 1.53	58.95 ± 1.15	62.09 ± 0.62
Carcass yield II (%)	60.93 ± 1.52	57.80 ± 1.16	60.95 ± 0.62

SE = standard error; means within the same row marked with different letters (a,b) differ significantly (*p* < 0.05); C, control; SMT, 2% non-fermented milk thistle; FMT, 1% fermented milk thistle + 1% substrate.

**Table 19:** Carcass yield at 77 days of age.

	C Average ± SE	SMT Average ± SE	FMT Average ± SE
Number of animals	11	14	7
Live weight (g)	2737.50 ± 23.03	2758.57 ± 14.74	2734.29 ± 44.71
Carcass weight (g)	1372.92 ± 18.23	1386.93 ± 15.41	1368.29 ± 36.79
Liver (g)	114.00 ± 2.93	118.07 ± 2.65	118.00 ± 3.13
Head (g)	125.33 ± 1.75	124.14 ± 0.96	123.57 ± 1.54
Kidney with fat (g)	35.58 ± 1.91	39.14 ± 1.07	38.57 ± 1.56
Carcass I (g)	1565.50 ± 20.84	1582.14 ± 16.58	1559.86 ± 38.70
Carcass II (g)	1647.83 ± 21.67	1668.29 ± 16.36	1648.43 ± 40.59
Carcass yield I (%)	61.34 ± 0.49	61.62 ± 0.39	61.32 ± 0.53
Carcass yield II (%)	60.18 ± 0.49	60.47 ± 0.38	60.24 ± 0.53

SE = standard error; C, control; SMT, 2% non-fermented milk thistle; FMT, 1% fermented milk thistle + 1% substrate.

## Data Availability

Data are available.
